# Mapping the Global Research on Drug–Drug Interactions: A Multidecadal Evolution Through AI-Driven Terminology Standardization

**DOI:** 10.3390/bioengineering12070783

**Published:** 2025-07-19

**Authors:** Andrei-Flavius Radu, Ada Radu, Delia Mirela Tit, Gabriela Bungau, Paul Andrei Negru

**Affiliations:** 1 Doctoral School of Biological and Biomedical Sciences, University of Oradea, 410087 Oradea, Romania; dtit@uoradea.ro (D.M.T.); gbungau@uoradea.ro (G.B.); negru.paulandrei@student.uoradea.ro (P.A.N.); 2 Department of Psycho-Neurosciences and Recovery, Faculty of Medicine and Pharmacy, University of Oradea, 410073 Oradea, Romania; 3 Department of Pharmacy, Faculty of Medicine and Pharmacy, University of Oradea, 410028 Oradea, Romania; 4 Department of Preclinical Disciplines, Faculty of Medicine and Pharmacy, University of Oradea, 410073 Oradea, Romania

**Keywords:** drug–drug interactions, drug interaction checker, bibliometric analysis, Python, VOSviewer, Web of Science

## Abstract

The significant burden of polypharmacy in clinical settings contrasts sharply with the narrow research focus on drug–drug interactions (DDIs), revealing an important gap in understanding the complexity of real-world multi-drug regimens. The present study addresses this gap by conducting a high-resolution, multidimensional bibliometric and network analysis of 19,151 DDI publications indexed in the Web of Science Core Collection (1975–2025). Using advanced tools, including VOSviewer version 1.6.20, Bibliometrix 5.0.0, and AI-enhanced terminology normalization, global research trajectories, knowledge clusters, and collaborative dynamics were systematically mapped. The analysis revealed an exponential growth in publication volume (from 55 in 1990 to 1194 in 2024), with output led by the United States and a marked acceleration in Chinese contributions after 2015. Key pharmacological agents frequently implicated in DDI research included CYP450-dependent drugs such as statins, antiretrovirals, and central nervous system drugs. Thematic clusters evolved from mechanistic toxicity assessments to complex frameworks involving clinical risk management, oncology co-therapies, and pharmacokinetic modeling. The citation impact peaked at 3.93 per year in 2019, reflecting the increasing integration of DDI research into mainstream areas of pharmaceutical science. The findings highlight a shift toward addressing polypharmacy risks in aging populations, supported by novel computational methodologies. This comprehensive assessment offers insights for researchers and academics aiming to navigate the evolving scientific landscape of DDIs and underlines the need for more nuanced system-level approaches to interaction risk assessment. Future studies should aim to incorporate patient-level real-world data, expand bibliometric coverage to underrepresented regions and non-English literature, and integrate pharmacogenomic and time-dependent variables to enhance predictive models of interaction risk. Cross-validation of AI-based approaches against clinical outcomes and prospective cohort data are also needed to bridge the translational gap and support precision dosing in complex therapeutic regimens.

## 1. Introduction

Pharmacological treatments are associated with a broad spectrum of clinical risks, including medication errors, adverse drug events, adverse drug reactions (ADRs), and drug–drug interactions (DDIs), which are collectively referred to as drug-related problems. The lack of universally accepted terminology and consistent definitions for these categories often hinders comparability between studies and impedes the calculation of incidence rates [1].

DDIs constitute one of the most pressing and resource-intensive challenges within healthcare systems, being implicated in a significant proportion of ADRs, with estimates indicating that they account for approximately 6% to 30% of all ADRs. Furthermore, DDIs are responsible for nearly 2.8% of hospital admissions associated with ADRs [2,3]. The likelihood of potential drug–drug interactions (pDDIs) rises substantially in settings characterized by polypharmacy, a condition typically described as the simultaneous daily administration of five or more medications to an individual [4].

The probability of pDDIs increases substantially with the number of medications administered concurrently, rising from an estimated 6% when two drugs are used to approximately 50% with five medications and nearly 100% when eight drugs are taken simultaneously [2]. Across various geographical regions, the reported prevalence rates of pDDIs exhibit considerable variability. In the United States, these rates have been observed to fall between 7.7% and 30.2%, whereas in European cohorts, estimates span from 0.8% up to 54.3%. In contrast, data from Australia indicate that roughly 1.5% of the elderly population may be at risk. Such discrepancies are likely influenced by heterogeneity in the population characteristics, including disease prevalence and pharmacological load, as well as methodological differences such as sample composition and study design [5].

Approximately six decades ago, the medical community, prompted by reports of life-threatening hypertensive episodes in individuals receiving monoamine oxidase inhibitors for depressive disorders following the ingestion of specific types of cheese, first began to acknowledge the clinical consequences of drug interactions. These adverse events were subsequently linked to the presence of high concentrations of tyramine, a pressor amine, whose intestinal degradation was markedly reduced due to monoamine oxidase inhibition. Around the same period, a separate pharmacokinetic interaction was described involving the concomitant use of the sulfonamide antibiotic sulphaphenazole and the antidiabetic agent tolbutamide, which resulted in hypoglycemic episodes. The underlying mechanism was attributed to a potential inhibition of tolbutamide metabolism by sulphaphenazole [6].

Drug interactions are generally categorized based on their underlying mechanisms into pharmaceutical, pharmacokinetic, and pharmacodynamic types. Pharmaceutical interactions, often referred to as pharmaceutical incompatibilities, occur when the precipitating substance influences the object drug prior to its administration [7]. Pharmacokinetic drug interactions commonly evaluate how one drug (i.e., the precipitant drug) alters the ADME processes of another by comparing these processes in the presence and absence of the interacting agent. In contrast, pharmacodynamic interactions are typically categorized as synergistic, additive, or antagonistic, depending on the observed modifications in the pharmacological effects of the drugs involved [8].

Patients diagnosed with cardiovascular diseases, where therapeutic regimens often involve multiple pharmacological agents, represent the group most frequently affected by clinically significant pDDIs [9]. Additionally, infectious diseases pose a high risk for drug interactions due to the susceptibility of antibiotics to such effects [3], particularly because of the frequent use of proton pump inhibitors [10,11]. A notable distinction exists between pDDIs and DDIs that are clinically significant. Therefore, cautious clinical judgment is essential prior to modifying treatment regimens based solely on pDDIs flagged by various software systems [12]. Recognizing the most clinically impactful DDIs within primary-care settings is critical for ensuring patient safety. Strategies to minimize the risk of DDIs include limiting the quantity of prescribed drugs, conducting frequent therapy evaluations, considering non-drug treatment options, closely observing signs of adverse effects or therapeutic outcomes, adjusting dosages when needed, and tailoring the timing of drug administration [13].

A wide range of scientific information resources support the dissemination and retrieval of drug-related knowledge across the biomedical research community. Among these, PubMed [14] and PubMed Central [15], maintained by the U.S. National Library of Medicine, offer open and indexed access to millions of peer-reviewed articles, including many free full-text manuscripts relevant to DDIs. Scopus [16], Web of Science [17], and Embase [18] further provide comprehensive indexing and citation data critical for bibliometric analyses and evidence synthesis. Full-text platforms such as ScienceDirect [19], ClinicalKey [20], and Wiley Online Library [21] further enable advanced literature navigation across medical and pharmaceutical domains, including the domain of DDIs. In parallel, authoritative regulatory databases hosted by agencies like the U.S. Food and Drug Administration [22] and the European Medicines Agency [23] offer structured drug interaction guidelines, labeling information, and pharmacovigilance data. These curated resources complement computational and AI-based tools by contextualizing their pharmacological mechanisms and clinical relevance. Their integration into both academic research and clinical workflows fosters informed decision-making and facilitates translational approaches to complex medication regimens. Moreover, several available online platforms are specifically designed to check for drug interactions, including Drugs.com^®^, Lexicomp^®^, Medscape^®^ [24], DDInter^®^ [25], WebMD, and DrugBank [3], with each providing advisory information intended to support clinical decision-making.

Certain drug interactions can be beneficial by improving their therapeutic effects through specific mechanisms. Synergistic pairings, such as ampicillin with gentamicin for enterococcal endocarditis, produce bactericidal effects unattainable by either drug alone, while tuberculosis treatment combining rifampin and isoniazid limits resistance development. Enhancement of drug levels occurs when ritonavir inhibits CYP3A4, thereby boosting the efficacy of darunavir [26] or lopinavir [7]. Beta-lactamase inhibitors like clavulanic acid aid amoxicillin by neutralizing bacterial resistance. Some combinations reduce toxicity; for instance, aluminum–magnesium hydroxide in antacids balances adverse reactions. Antidotal use of folic acid counters methotrexate toxicity. Combinations like carbidopa–levodopa provide dual benefits by increasing efficacy and lowering side effects, as carbidopa prevents peripheral dopamine conversion, minimizing systemic toxicity while preserving its central action [7].

The rapid growth in studies of drug interactions has occurred despite the fact that research has focused almost exclusively on combinations of two drugs, whereas in real-world prescribing scenarios, patients are often treated with ten or more drugs concomitantly. This expanding body of literature highlights the need for systematic bibliometric analyses to track scientific trends, identify influential publications, and evaluate key bibliometric parameters such as publication volume, citation impact, and collaborative networks. Such analyses provide valuable insights into methodological evolution and thematic trends, thereby serving as a critical tool for advancing scientific knowledge and assessing the state of research across pre-research, basic, and applied stages. However, bibliometric data on DDIs remain scarce and fragmented, with limited attempts to systematically map the intellectual, thematic, and collaborative evolution of this critical research field. Given the escalating global burden of polypharmacy, the scarcity of comprehensive bibliometric assessments represents a research gap.

Our research aims to provide a high-resolution, multidimensional mapping of global DDI research through a comprehensive bibliometric and network analysis, with a focus on identifying structural shifts, knowledge clusters, and the most relevant bibliometric parameters. The contribution lies not only in the scale of the dataset and the limited bibliometric approaches in this field but also in the integration of artificial intelligence (AI)-driven terminology normalization, enabling high accuracy in thematic classification.

## 2. Materials and Methods

The present bibliometric assessment utilized the Web of Science Core Collection as the primary data repository for comprehensive literature retrieval. The selection of this database was strategically motivated by several methodological considerations to ensure robust analytical outcomes. The Web of Science Core Collection provides extensive multidisciplinary coverage spanning the pharmaceutical sciences, clinical medicine, pharmacology, and toxicology domains essential for DDI research. The database maintains high-quality bibliographic records with comprehensive metadata, including complete author affiliations, citation networks, and keyword indexing systems that facilitate advanced bibliometric mapping techniques. Furthermore, the database’s rigorous peer-review standards and established indexing protocols ensure the inclusion of high-impact, methodologically sound research publications. The consistency of data formatting and standardized field structures within the Web of Science Core Collection minimizes technical complications during data processing and enhances the reliability of cross-reference analysis. By utilizing a single, comprehensive database, methodological complexities associated with multi-database integration were circumvented, thereby strengthening the reproducibility and validity of the analytical framework [27].

**Figure 1:**
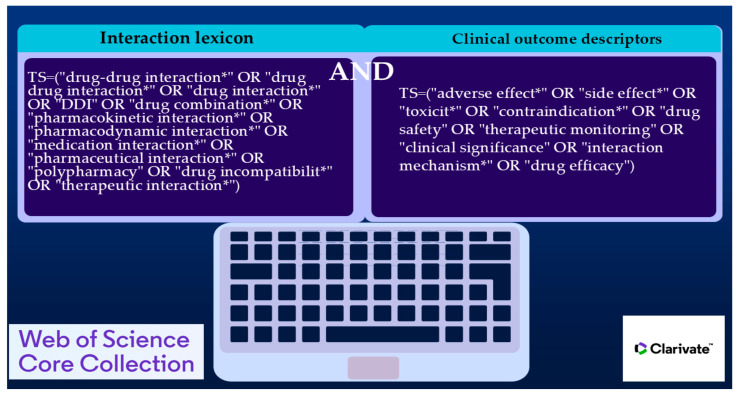
Primary search query used in the Web of Science database.

An extensive search strategy was developed to thoroughly explore the literature on DDIs while ensuring high precision in the results. This approach was based on two main concepts: terminology related to interactions and terms describing clinical outcomes. By combining these elements, the search effectively captured both the mechanistic aspects and the practical implications of DDI research. The primary search query is displayed descriptively in Figure 1 [28].

To enhance specificity and minimize false positives, the Boolean operator “AND” was used to ensure that retrieved documents addressed both drug interaction mechanisms and their clinical relevance. Within each conceptual group, the “OR” operator broadened the search by including synonyms and alternative expressions frequently used in various medical disciplines and research settings. Truncation symbols (e.g., *) were strategically used to account for different word forms, such as singular, plural, and related derivatives of core terms. This method increased the sensitivity of the search by retrieving a wider range of relevant records while still aligning with the central concepts of the research area.

**Figure 2:**
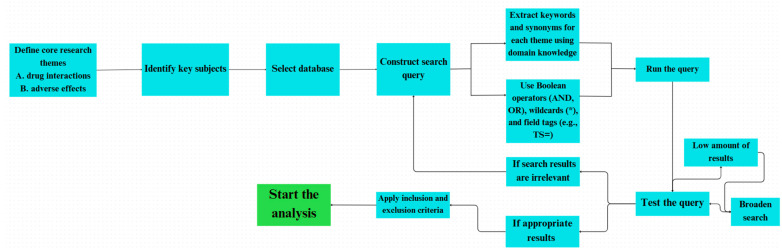
Flow diagram illustrating the screening and selection process for literature retrieved from the Web of Science database.

The initial search execution yielded 20,710 documents spanning the complete temporal range available in the Web of Science database. To optimize the dataset quality and analytical consistency, a systematic filtering protocol was implemented according to predetermined inclusion criteria (Figure 2).

The inclusion criteria encompassed exclusively articles and reviews published in the English language, ensuring linguistic consistency, essential for subsequent keyword analysis and thematic interpretation. Application of these criteria resulted in a refined dataset of 19,151 documents, representing a 7.5% reduction, which significantly enhanced the data quality while preserving comprehensive coverage of the research domain. This filtering approach ensured linguistic homogeneity, essential for accurate keyword co-occurrence analysis, and eliminated publication types with limited citation potential that could skew bibliometric indicators.

A comprehensive visualization assessment of collaborative networks and the thematic evolution was facilitated through the implementation of a bibliometric analysis that employed multiple integrated platforms utilizing diverse, mutually reinforcing software applications. Network visualizations were primarily generated using VOSviewer (version 1.6.20) [29], which served as the principal instrument for constructing bibliometric maps that depicted keyword co-occurrence structures and research collaboration patterns at the country level. The R statistical environment’s Biblioshiny web interface [30] provided access to the Bibliometrix package (version 5.0.0) [31], which was utilized to conduct trend analysis in the field of DDIs. Data preprocessing, descriptive statistics generation, and the creation of supplementary visualizations were accomplished utilizing Microsoft Excel (Microsoft Office Professional Plus 2019), thereby improving the interpretability and clarity of intricate bibliometric results.

The significant discrepancy technically observed between China’s publication counts from VOSviewer) and Bibliometrix reflects different counting methodologies employed by these bibliometric tools. VOSviewer uses fractional counting, dividing credit for international collaborations among participating countries, while Bibliometrix applies full counting, crediting each country fully for collaborative publications. This difference is particularly significant for China, given its substantial increase in international research partnerships since 2015. Fractional counting provides a more conservative estimate of unique national contributions, while full counting captures the complete scope of research participation.

Annual publication trends across leading countries were visualized using Python 3.12.3 libraries, including Pandas version 2.3.1 [32], Matplotlib version 3.10.3 [33], Seaborn version 0.13.2 [34], and NumPy version 2.3.1 [35]. Cumulative publication data were converted to annual increments and organized in country-by-year matrices. Heat maps were created using a yellow–orange–red colormap to represent publication intensity, with grid lines for clarity and 5-year-interval labeling on the *x*-axis. This approach facilitated identification of temporal research patterns and national productivity trends across the 51-year study period.

Total link strength (TLS) is a VOSviewer metric representing the cumulative strength of a node’s connections with all other nodes in the network. It was employed to quantify collaborative relationships and keyword associations.

The analysis of country collaboration networks incorporated nations contributing a minimum of 5 publications to establish meaningful collaboration patterns while excluding countries with an insufficient publication volume for reliable network positioning. Node dimensions in the network visualization reflected total publication counts, with larger nodes representing nations demonstrating greater research productivity. The strength of collaborative relationships between countries was indicated through the line thickness, which served as a visual representation of both the frequency and intensity of international research partnerships. Data normalization was achieved through manually identifying different spellings of the same country (i.e., Turkiye and Turkey).

The thematic evolution and temporal distribution analyses were conducted using the Bibliometrix package (version 5.0.0) through its specialized functions for longitudinal bibliometric assessment. Thematic evolution was examined using the thematic evolution function of the Biblioshiny web interface, which generated Sankey diagrams to visualize the transformation and flow of research themes across three predefined temporal segments (1975–2000, 2001–2010, and 2011–2025), with inclusion thresholds set to capture themes appearing in at least 50 documents per period to ensure statistical relevance. The temporal distribution of the research terminology was analyzed using the trend topics, enabling the identification of temporal patterns in keyword emergence and their persistence throughout the dataset.

Network visualizations for keyword co-occurrence analysis included terms appearing in at least 100 publications, ensuring statistical significance while capturing the comprehensive breadth of research terminology within the field. Node proximity in these visualizations was determined by the co-occurrence frequency, with closely positioned terms indicating regular simultaneous usage throughout the literature, while VOSviewer’s association strength calculations enabled the identification of thematically related keyword clusters through systematic color-coding.

**Figure 3:**
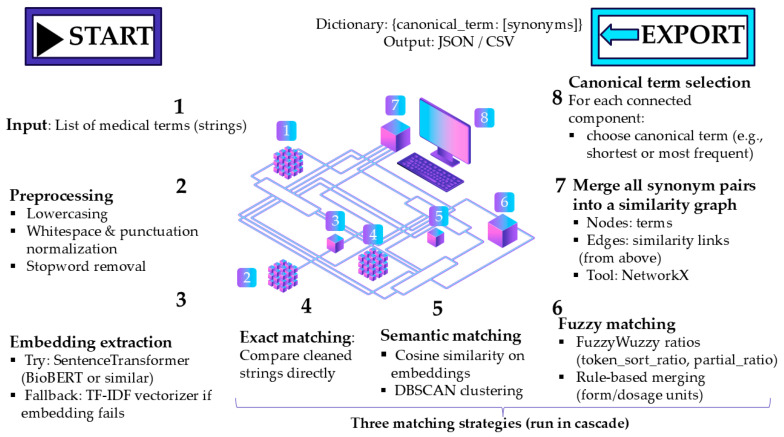
System architecture of the medical-term normalization and synonym resolution pipeline. The process begins with a list of input terms, which are preprocessed and vectorized. Matching is performed using a cascade of exact, semantic, and fuzzy strategies. Detected synonym pairs are merged into a graph structure from which canonical forms are selected per component. The final output is a dictionary mapping canonical terms to their variants.

A multi-method AI-enhanced system for automated synonym detection and terminology standardization in pharmaceutical literature has been developed (437 lines of Python code) that is specifically designed for DDI research vocabularies. The system employs a three-tiered approach combining exact character variation detection using regular expressions to identify formatting inconsistencies, semantic similarity clustering using pre-trained transformer models (BioBERT and SentenceTransformers) with DBSCAN clustering (cosine similarity threshold ≥0.85), and medical context-aware fuzzy string matching (FuzzyWuzzy library) that considers pharmaceutical components such as dosage forms, concentrations, and active ingredients [36]. The architecture of the system (Figure 3) follows a structured pipeline: after the initial term cleaning and validation, the three modules—exact matching, semantic embedding-based clustering, and fuzzy rule-based matching—operate in parallel. Their outputs are subsequently merged using a graph-based resolution mechanism, where overlapping synonym groups are unified, and canonical terms are selected through component-wise analysis. The final output is a normalized term dictionary that is optimized for downstream text mining and information retrieval in biomedical domains.

The algorithm incorporates domain-specific validation patterns for medical terminology, handles missing dependencies through graceful fallbacks, and uses graph-based merging (NetworkX) to resolve overlapping synonym groups while maintaining precision through minimum group-size constraints. Input terms undergo preprocessing to normalize punctuation and to extract core pharmaceutical components, with the final output formatted as comma-separated value files that are compatible with VOSviewer visualization software, enabling standardized thesaurus integration for bibliometric and systematic review applications. The system’s precision was validated using a stratified random sample of 300 term pairs from the generated thesaurus. Three domain experts in pharmaceutical sciences independently evaluated each synonym grouping as correct or incorrect based on semantic equivalence criteria. The system’s performance was deemed acceptable only when all three evaluators unanimously agreed that ≥90% of the groupings were correct.

To clarify the role of AI in our methodology, we distinguish between two phases of our analysis. In the first phase, we use AI/machine learning techniques for terminology standardization. We utilize transformer-based language models (BioBERT) and unsupervised clustering algorithms (DBSCAN with a cosine similarity threshold of ≥0.85) to intelligently identify semantic relationships between different terms. This AI-driven pre-processing step is crucial for overcoming the significant challenge of keyword variability in bibliometric studies, where authors may use dozens of variations to describe the same concept. The second phase applies traditional bibliometric and data-mining techniques, which include publication counting, citation analysis, and network visualization, to the standardized dataset. While these established methods do not constitute AI, the quality and accuracy of their outputs are substantially enhanced by the initial AI-driven terminology normalization. This enables a more reliable identification of research trends and thematic clusters than would be possible with conventional keyword matching alone.

## 3. Results

### 3.1. Scientific Literature Overview

**Figure 4:**
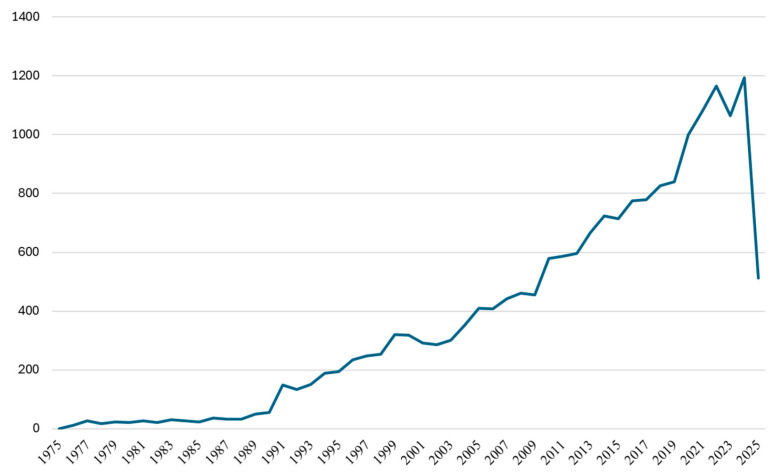
Temporal distribution of scientific publications corresponding to the implemented search algorithm.

The bibliometric analysis covers a total of 19,151 publications over a 51-year period (1975–2025), highlighting the significant evolution of scientific interest in drug–drug interactions and pharmaceutical safety. This extensive dataset reflects the field’s development from its early stages to its current prominence as a vital area within contemporary pharmacotherapy research. The temporal distribution of publications (Figure 4) reveals four distinct developmental phases, each characterized by unique growth dynamics, that reflect broader changes in pharmaceutical science, regulatory frameworks, and clinical practice priorities.

The earliest phase represents the foundational years of systematic drug–drug interaction research, which are characterized by minimal but consistent scientific output. Annual publication volumes remained below 60 documents throughout this period, beginning with a single publication in 1975 and gradually increasing to 55 articles by 1990. In 1991, the field experienced a significant paradigm shift, as evidenced by a sharp rise in the publication output from 55 to 149 articles, representing a 171% increase. This surge underscored the growing awareness of drug–drug interactions as an increasingly important issue in clinical practice. The third phase was characterized by a period of relative stability, with annual publication counts ranging between 286 and 461 articles. Although it lacked sharp growth spikes, this decade reflected a consistent upward trend, indicating sustained research interest in the field. The most recent phase demonstrates renewed acceleration, beginning with 580 publications in 2010 and culminating in a record-breaking level of productivity with 1194 articles in 2024. This remarkable 106% increase over 14 years reflects the integration of drug–drug interaction research with emerging scientific paradigms.

**Figure 5:**
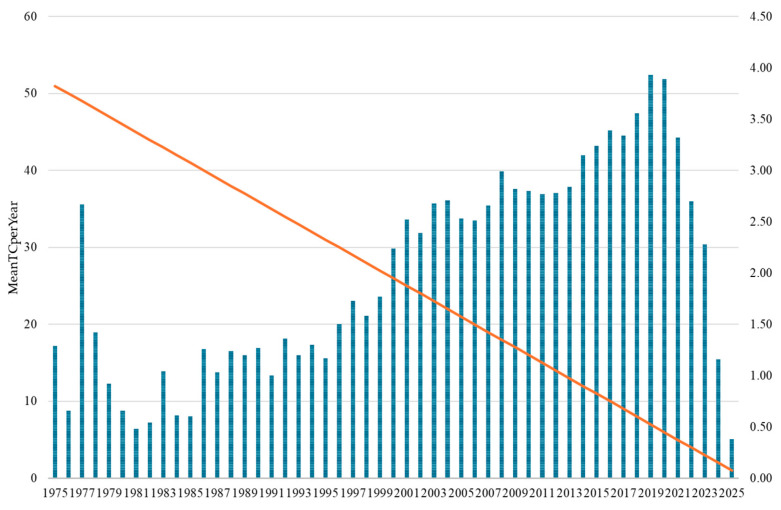
Temporal trends in mean annual citations reflecting the scientific maturation and impact of drug–drug interaction research (1975–2025).

The temporal evolution of citation patterns (Figure 5) reveals the progressive scientific maturation and influence of drug–drug interaction research across five decades. Mean Total Citations per Year (MeanTCperYear) demonstrated a clear upward trajectory, beginning with modest values of below 2.0 during the foundational period (1975–1990), gradually increasing through the second phase of the 1990s (1.00–1.77), and achieving a substantial impact during the early 2000s period (2.24–2.99). Between 2014 and 2019, the field reached its peak for citation impact, with the highest rate recorded in 2019 at 3.93 citations per year. Although citation rates declined from 3.89 to 0.38 between 2020 and 2025, this trend largely reflects the typical citation lag for more recent publications rather than a drop in research quality. Notably, publications from 2020–2021 maintained high citation levels, highlighting ongoing strong scientific interest, especially in the context of Coronavirus disease 2019 (COVID-19)-related drug interaction studies. This citation evolution demonstrates the field’s transformation from a specialized pharmacological concern to a central component of evidence-based medicine, with contemporary research achieving a remarkable influence on clinical practice and pharmaceutical development.

### 3.2. Academic Influence and Impact

The analysis of global contributions to drug–drug interaction (DDI) research (Table 1) reveals a pronounced geographic concentration, with the United States leading the field. The U.S. accounts for 7001 publications (36.6% of the total) and demonstrates its strong influence with a high average citation rate of 41.11 per document; in addition, it has the most extensive international collaboration network, as shown by a TLS of 2610. Canada stands out for its research efficiency, producing the highest citation impact worldwide (46.32 citations per document) despite being sixth in output with 846 publications. The United Kingdom also shows a strong profile, combining high productivity (1404 publications) with significant citation influence (40.22 citations per document).

Several European countries, including Italy (1124 publications), Germany (1045), and France (819), exhibit well-rounded research performances marked by both output and robust international collaboration. In contrast, Asian countries present varied profiles: China leads in volume among the Asian nations (1723 publications) but lags in citation impact (22.85 citations per document), while India shows growing research engagement (818 publications) with a lower impact (17.73). Japan maintains a more balanced performance, with 664 publications and a moderate citation impact (25.32).

**Figure 6:**
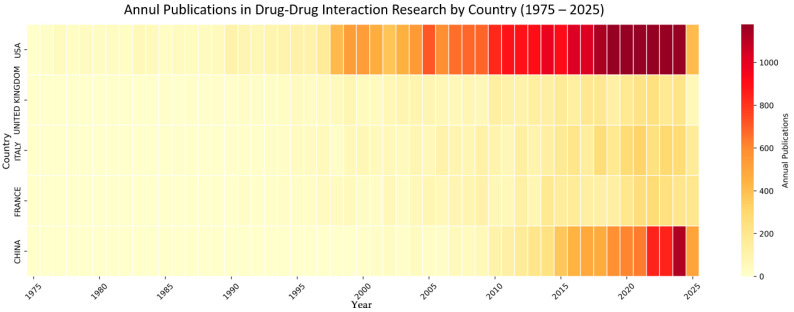
Heat map of global research leadership dynamics in drug–drug interaction studies (1975–2025).

Figure 6 presents a temporal heatmap visualization that tracks the annual publication output from the five most productive countries in DDI research from 1975 to 2025. The United States established early and sustained dominance, beginning with just 2 publications in 1975 and expanding rapidly to a cumulative total of 24,650 by 2025. European countries entered the field gradually—the United Kingdom in 1977, Italy in 1978, and France in 1980, with each following a steady growth trajectory supported by well-established pharmaceutical research infrastructure and regional coordination. By the year 2000, the research landscape had stabilized, with the United States in the lead (2816 publications), followed by the UK (405), Italy (361), and France (283). China, who entered the field relatively late with a single publication in 1991, remained marginal contributor through the early 2000s.

However, a major shift occurred after 2015, when China experienced a dramatic surge in output, growing from 1497 publications in 2015 to 8037 by 2025. This more than fivefold increase enabled China to surpass France (in 2017), the United Kingdom (in 2019), and Italy (in 2020), firmly establishing itself as the second-largest contributor to global DDI research. By 2025, the global hierarchy had shifted: the United States retained its leading position (24,650 publications), followed by China (8037), with Italy (4139), the UK (3606), and France (3463) trailing behind with more gradual growth.

### 3.3. Scientific Impact Analysis

Analysis of publication sources (Table 2) reveals a diverse and multifaceted landscape, with *Clinical Pharmacokinetics* emerging as the leading specialized journal in the field. Since its establishment in 1977, the journal has become a key platform for drug–drug interaction research, publishing 158 articles and achieving notable h- and m-indices of 71 and 1.449, respectively. By contrast, the *Journal of Clinical Oncology*, despite having a broader clinical scope, is notable for its exceptional citation efficiency, averaging 137.3 citations per document. These differences emphasize the dual nature of the field, where both niche pharmacological journals and high-impact clinical outlets play pivotal roles in disseminating influential research. The top ten journals represent a balanced distribution between specialized pharmacology venues (*Drug Safety*, *Clinical Pharmacology & Therapeutics*, *British Journal of Clinical Pharmacology*) and clinical specialty publications (*Clinical Cancer Research*, *Epilepsia*) alongside practice-oriented platforms (*Annals of Pharmacotherapy*, with the highest volume at 274 documents; *American Journal of Health-System Pharmacy*). Notable patterns include *Clinical Cancer Research*, which achieved the highest m-index (1.633), indicating exceptional impact efficiency, while *Drugs* demonstrated remarkable citation efficiency (91.0 citations per document) through comprehensive review publications and *Drug Safety* maintained the second-highest m-index (1.457), reflecting sustained research quality since 1991.

**Figure 7:**
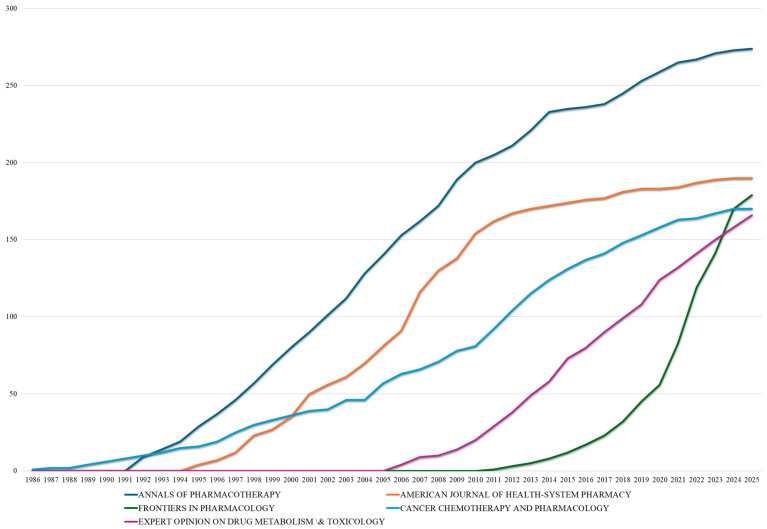
Temporal trends in leading journal contributions to drug–drug interaction research (1986–2025).

The temporal analysis of leading journals in DDI research (Figure 7) reveals evolving publication trends that mirror shifting research priorities and the emergence of new dissemination platforms. *Annals of Pharmacotherapy* showed the most substantial overall growth, starting with 9 publications in 1992 and reaching 274 by 2025. However, its recent growth slowed significantly (from 259 in 2020 to 274 in 2025), suggesting a saturation point within pharmacy-practice-focused DDI research. Similarly, the *American Journal of Health-System Pharmacy* followed a steady upward trajectory from its entry in 1995, reaching 183 publications by 2020 before leveling off at 190 by 2025, indicating a maturation of its role in the field.

The most striking trend was the rapid rise of *Frontiers in Pharmacology*, which entered the DDI landscape in 2011 with just 1 publication but which surged to 179 by 2025. This dramatic ascent underscores the growing influence of open-access publishing in accelerating the visibility and dissemination of research. *Cancer Chemotherapy and Pharmacology*, in contrast, displayed the most consistent long-term growth, beginning in 1986 and reaching 170 publications by 2025, reflecting the steady integration of DDI considerations into oncology.

**Figure 8:**
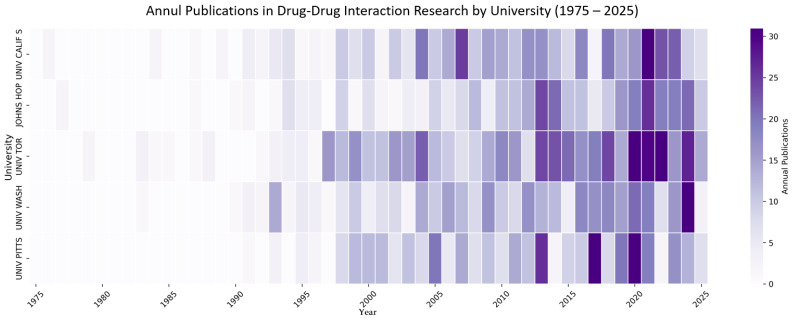
Heat map of institutional growth trajectories in drug–drug interaction research (1975–2025).

Analysis of institutional research output (Figure 8) revealed distinct growth trajectories among the leading academic centers in drug–drug interaction research. The University of Toronto emerged as the most prolific institution, demonstrating exceptional growth from just 2 publications in 1979 to 538 cumulative publications by 2025, representing a 269-fold increase. The University of Washington followed a similar but more pronounced trajectory, starting with its first publication in 1983 and reaching 390 publications by 2025. Notably, both institutions experienced critical acceleration phases: the University of Toronto showed its first major growth surge between 1997–1999 (from 40 to 69 publications), while the University of Washington’s output intensified dramatically after 2000, jumping from 53 to 299 publications between 2000 and 2020.

The data reveal three distinct phases of institutional productivity evolution. The initial phase (1975–1990) was characterized by minimal activity, with only the University of California San Francisco and Johns Hopkins University maintaining a modest presence. The expansion phase (1991–2005) marked a turning point when all five institutions began substantial research programs, with cumulative publications increasing by an average of 10–15 papers annually. The acceleration phase (2006–2025) witnessed unprecedented growth, particularly for the University of Pittsburgh, which, despite entering the field the latest (1991), achieved remarkable momentum, surpassing both UCSF and Johns Hopkins to reach 375 publications. This institutional analysis underscores how drug–drug interaction research has evolved from a niche field dominated by a few pioneers to a rapidly expanding domain with multiple centers of excellence, each contributing substantially to the knowledge base.

The examination of highly cited publications (Table 3) in drug–drug interaction research reveals significant temporal and thematic diversity, with citation counts ranging from 793 to 2743, demonstrating the substantial academic impact of foundational research in this field. The most influential work by Gaede et al. (2008) in the *New England Journal of Medicine* (2743 citations) established paradigms for multifactorial therapeutic interventions in diabetes management, while Goldberg et al. (2004), in the *Journal of Clinical Oncology* (1877 citations), revolutionized combination chemotherapy protocols through the systematic evaluation of drug interaction profiles. The temporal distribution spans from pioneering combination therapy research by Einhorn and Donohue (1977) to contemporary mechanistic investigations, with notable contributions from Lynch and Price (2007) on cytochrome P450-mediated interactions and Scott et al. (2015) on evidence-based deprescribing methodologies. The journal diversity encompasses prestigious clinical publications (*NEJM*, *JAMA Internal Medicine*), specialized research venues (*Nature Reviews Clinical Oncology*, *Free Radical Biology and Medicine*), and methodological resources (*Nucleic Acids Research*), reflecting the interdisciplinary nature of contemporary drug interaction research. These influential works have collectively transformed clinical practice from reactive drug interaction management to proactive, evidence-based polypharmacy optimization, establishing foundational principles that continue to guide therapeutic decision-making across multiple medical specialties.

### 3.4. Scientific Mapping

**Figure 9:**
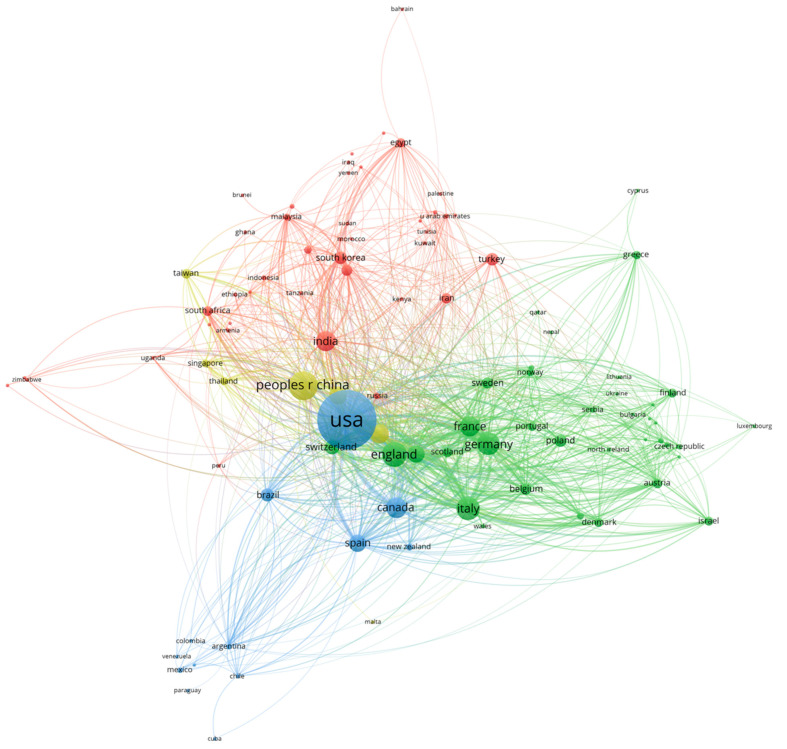
Global collaboration network in DDI research (1975–2025).

The global collaboration network (Figure 9) in DDI research reveals four distinct regional clusters that reflect both geographical proximity and shared research priorities across 100 contributing countries. The largest cluster (red) represents a diverse Asia–Africa–Middle East network encompassing 43 countries including India (401 TLS), Saudi Arabia (266 TLS), South Korea (240 TLS), Turkey (130 TLS), Egypt (181 TLS), and Iran (87 TLS), demonstrating extensive collaboration among emerging pharmaceutical markets with shared challenges in drug safety evaluation and regulatory development. The green cluster comprises 37 countries forming a primarily European research establishment, centered on England (1491 TLS), Germany (1057 TLS), Italy (979 TLS), and France (831 TLS), representing the most densely connected collaborative network with mature research infrastructure and established regulatory frameworks. The Americas-centered blue cluster includes 13 countries dominated by the U.S.’s extensive international partnerships (2611 TLS) and significant collaborations with Canada (680 TLS), Brazil (240 TLS), and Spain (727 TLS), reflecting both North–South knowledge transfer and shared regulatory approaches. Finally, the yellow cluster represents the Asia–Pacific hub with 7 countries, centered on China (688 TLS), Japan (356 TLS), Australia (592 TLS), and Singapore (207 TLS), indicating a focused regional pharmaceutical research ecosystem. This network structure demonstrates that while established research centers maintain a high collaboration intensity, the largest collaborative cluster now encompasses the global South, reflecting the democratization of pharmaceutical safety research and the emergence of diverse regional approaches to drug–drug interaction studies tailored to population-specific needs and healthcare contexts.

**Figure 10:**
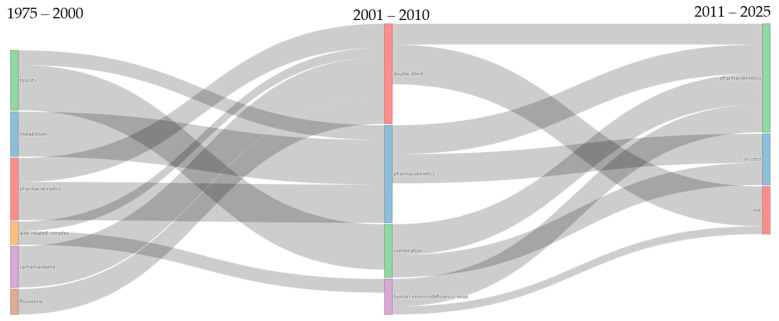
Thematic evolution map of DDI research (1975–2025), illustrating the emergence, merging, and persistence of key thematic clusters across three distinct time periods.

The thematic evolution analysis revealed a clear transformation in DDI research across three distinct periods (Figure 10). During 1975–2000, the field was characterized by six dispersed themes: toxicity (the largest cluster), metabolism, pharmacokinetics, AIDS-related complex, carbamazepine, and fluoxetine. The intermediate period (2001–2010) witnessed significant consolidation into four major themes: double-blind (receiving substantial flows from carbamazepine, fluoxetine, and AIDS-related complex), pharmacokinetics (maintaining strong continuity from the previous period), combination (evolving primarily from toxicity), and human-immunodeficiency virus (transforming from AIDS-related complex). The most recent period (2011–2025) demonstrated further specialization into three core domains: pharmacokinetics (showing remarkable persistence across all periods and receiving flows from both earlier pharmacokinetics and combination themes), in vitro studies (i.e., emerging from the convergence of combination and pharmacokinetics research), and risk assessment (i.e., developing from the integration of double-blind, human immunodeficiency virus, and pharmacokinetics themes).

**Figure 11:**
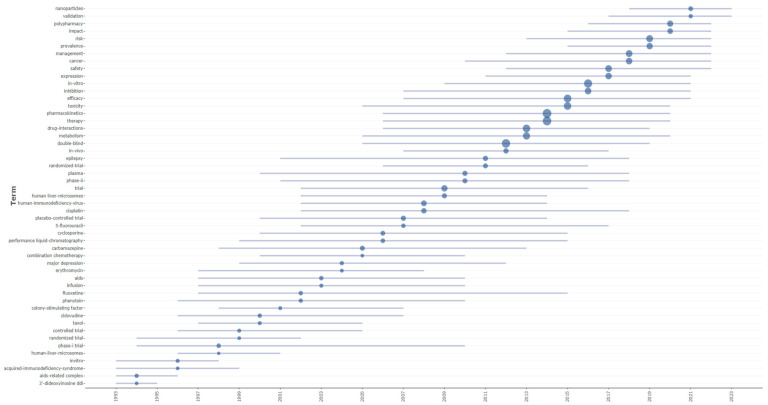
Temporal distribution of key research terms in the DDI literature by median publication year.

The temporal distribution of research terminology revealed distinct waves of thematic focus in DDI literature (Figure 11). Early research (1991–2000) was dominated by HIV/AIDS-related terminology, with terms such as “immune-deficiency syndrome” (median year: 1991), “azidothymidine azt” (median: 1993), and “AIDS-related complex” (median: 1994) representing the initial urgent response to the HIV epidemic. Figure 10 illustrates this temporal distribution, positioning keywords along a timeline based on their median publication year and representing their frequency by bubble size. This visualization clearly demonstrates the evolution from early disease-focused terminology through methodological standardization to contemporary technological innovations. A methodological transition occurred during 2000–2015, which was marked by the emergence of clinical trial terminology including “controlled trial” (median: 1999), “placebo-controlled trial” (median: 2007), and “double-blind” (median: 2012; frequency: 1364), alongside core scientific concepts like “pharmacokinetics” (median: 2014; frequency: 1559) and “drug-interactions” (median: 2013; frequency: 998). The most recent period (2016–2025) demonstrated a shift toward clinical implementation and emerging technologies, with “polypharmacy” (median: 2020; frequency: 456), “risk” (median: 2019; frequency: 725), and “management” (median: 2018; frequency: 654) indicating a focus on practical applications, while “nanoparticles” (median: 2021), “molecular docking” (median: 2023), and “nanomedicine” (median: 2024) represent cutting-edge technological approaches to DDI research.

**Figure 12:**
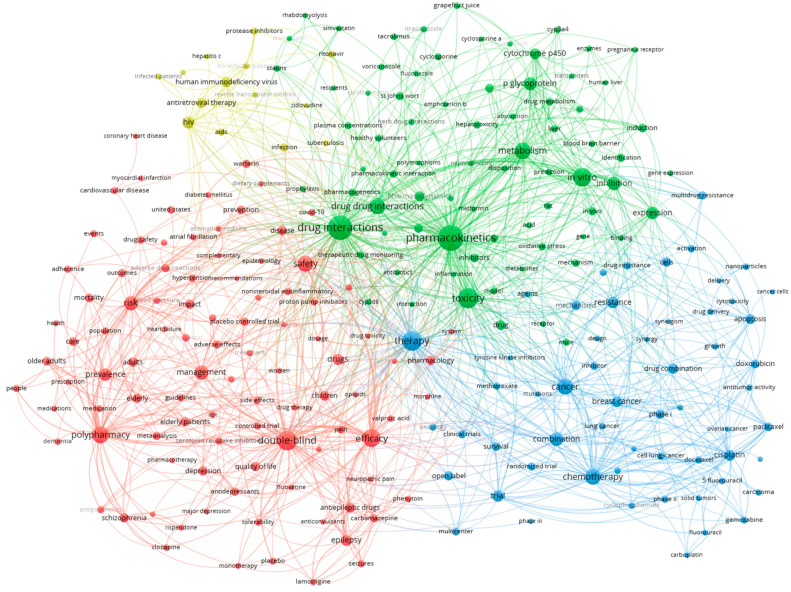
The keyword co-occurrence network of DDI-related terms.

The keyword co-occurrence network reveals a sophisticated research ecosystem comprising four distinct thematic domains that collectively define the DDI research landscape (Figure 12). The central positioning of “drug–drug interactions”, “drug interactions”, and “pharmacokinetics” as major hubs demonstrates their foundational role in connecting diverse research themes, while the substantial node sizes of terms such as “safety,” “metabolism”, and “chemotherapy” indicate their prominence in contemporary literature. The red cluster emphasizes clinical risk management and patient safety considerations, particularly highlighting the research focused on vulnerable populations, including elderly patients and polypharmacy scenarios. Meanwhile, the green cluster represents the mechanistic foundation of the field, with cytochrome P450 enzymes, drug metabolism, and pharmacokinetic processes forming a tightly interconnected knowledge network that underpins interaction prediction and assessment.

The network topology illustrates the field’s evolution toward interdisciplinary integration, where basic pharmacological mechanisms increasingly inform clinical decision-making and safety protocols. The strong connectivity between the blue therapeutic cluster (encompassing cancer treatment and chemotherapy) and the mechanistic green cluster suggests that oncology has emerged as a critical domain for drug interaction research, likely due to the complex polypharmacy regimens characteristic of cancer care. Additionally, the positioning of terms related to “therapeutic monitoring”, “clinical significance”, and “drug combinations” as bridging concepts between clusters indicates the field’s progression toward evidence-based interaction management strategies. This interconnected structure demonstrates that contemporary drug interaction research transcends traditional disciplinary boundaries, integrating computational prediction models, clinical evidence, and regulatory considerations into a unified approach for optimizing therapeutic outcomes while minimizing adverse effects.

## 4. Discussion

This comprehensive bibliometric analysis spanning five decades (1975–2025) reveals the transformation of drug–drug interaction research from a peripheral pharmacological concern to a central pillar of pharmaceutical safety. The exponential growth trajectory, particularly the increase during 2010–2024, reflects not merely quantitative expansion but fundamental shifts in research paradigms, methodological sophistication, and clinical integration. The five-decade evolution reveals exponential growth patterns consistent with emerging scientific disciplines achieving mainstream recognition. The total accumulation of 19,151 publications represents substantial intellectual investment in understanding and predicting drug–drug interactions, transitioning from isolated clinical observations to a comprehensive mechanistic understanding supported by sophisticated analytical frameworks. This sustained momentum indicates that drug–drug interaction research has achieved mature scientific status while continuing to evolve in response to new therapeutic challenges and technological capabilities, with 2024 representing the most productive year in the field’s history.

While early combination therapy successes like Einhorn and Donohue’s (1977) groundbreaking regimen combining cisplatin, vinblastine, and bleomycin for testicular cancer [37] demonstrated the power of multi-drug approaches, they also underscored the critical need to understand DDIs. This recognition led to systematic investigations of drug metabolism, particularly through cytochrome P450 pathways, which became the foundation of modern drug interaction science.

Throughout the 1990s and early 2000s, the research focus shifted toward a mechanistic understanding of specific drug–drug interactions, particularly cytochrome P450-mediated interactions. Key studies documented critical CYP3A4 interactions, such as the profound effects of ketoconazole, itraconazole, and macrolide antibiotics (clarithromycin, erythromycin) on drugs like terfenadine, cisapride, and statins [38].

The emergence of HIV-protease-inhibitor therapy revealed complex interaction networks, with ritonavir identified as both a potent CYP3A4 inhibitor and inducer, dramatically affecting concentrations of co-administered drugs including saquinavir, indinavir, and immunosuppressants like cyclosporin [39].

Antidepressant polypharmacy research highlighted significant interactions between selective serotonin reuptake inhibitors, particularly fluoxetine and paroxetine as CYP2D6 inhibitors affecting the metabolism of beta-blockers and tricyclic antidepressants [40,41].

Studies on statin interactions demonstrated that simvastatin and lovastatin, as CYP3A4 substrates, resulted in an increased risk of myopathy when combined with inhibitors like cyclosporine A and mibefradil [42,43]. This period established fundamental pharmacokinetic principles, including the recognition of drug-transporter involvement (particularly of P-glycoprotein) alongside CYP450 metabolism, that continue to guide drug interaction assessment today.

A paradigm shift occurred in the 2010s that was marked by the transition from studying isolated drug pairs to addressing complex polypharmacy scenarios. Gnjidic et al. established in 2012 that five or more medications significantly increased the risks of frailty, disability, and falls in elderly patients, with every additional medication increasing the odds of adverse outcome [44].

Supporting this computational evolution, DrugBank 4.0 expanded to include over 1200 drug metabolites and 1300 drug metabolism reactions, providing comprehensive ADMET data and tools for analyzing drug-target–enzyme-transporter associations to predict drug–drug interactions [45]. Real-world drug interaction data revealed critical safety signals through computational approaches, with Tatonetti et al. developing data-driven methods that identified novel interactions, including the QT prolongation risk with combined selective serotonin reuptake inhibitors and thiazides [46].

Clinical complexity was exemplified in HIV care, where Smit et al. projected that by 2030, 40% of HIV patients could face complications with first-line regimens due to drug–drug interactions from multiple comorbidities [47]. The management of complex therapies illustrated these challenges, as shown in the chronic pulmonary aspergillosis guidelines by Denning et al., which emphasized the careful monitoring of azole serum concentrations and drug interactions [48]. Deprescribing emerged as essential, with Scott et al. providing the following five-step protocol to systematically reduce inappropriate polypharmacy: identifying all current medications and their indications, evaluating the patient-specific risk of drug-related harm to guide intervention intensity, balancing each drug’s benefits against potential harms, prioritizing the cessation of drugs with minimal benefit and low withdrawal risk, and executing a tapering plan with close monitoring for clinical changes or adverse events [49]. This evolution culminated in Zitnik et al.’s (2018) graph convolutional networks (i.e., Decagon) for modeling polypharmacy side effects, which could predict the exact side effects of drug combinations and achieved up to 69% performance improvement over baselines, representing the field’s maturation from documenting interactions to developing comprehensive predictive frameworks [50].

The network topology reveals therapeutically coherent clustering patterns where disease-specific medication groups naturally aggregate, together with their associated safety concerns. Within the red cluster, three distinct therapeutic subclusters emerge, each with characteristic co-occurrence patterns. The epilepsy-focused subcluster demonstrates strong associations between seizures, antiepileptic drugs, and specific agents such as lamotrigine and carbamazepine, reflecting the well-documented interaction potential of these narrow-therapeutic-index medications. The psychiatric medication subcluster, also positioned within the red cluster, centers around schizophrenia, with the closely linked antipsychotics risperidone and clozapine, indicating concentrated research attention on psychotropic drug interactions. The demographic risk subcluster completes the red cluster’s composition, revealing polypharmacy as strongly associated with elderly and older adults, highlighting this population’s heightened vulnerability to interaction-related adverse events due to age-related pharmacokinetic changes and medication complexity.

Within the blue cluster, three distinct therapeutic subclusters emerge, each with characteristic co-occurrence patterns. The oncology-focused subcluster dominates, demonstrating dense interconnections between cancer types (i.e., breast, lung, and ovarian), chemotherapeutic agents (i.e., cisplatin, paclitaxel, doxorubicin, carboplatin, gemcitabine, and 5-fluorouracil), and treatment modalities (i.e., chemotherapy and combination therapy). This reflects the critical importance of understanding drug interactions in multi-agent cancer treatment protocols, where therapeutic windows are narrow and managing toxicity is paramount. The drug-resistance mechanisms subcluster, also positioned within the blue cluster, centers around multi-drug resistance, drug resistance, and cellular mechanisms, including apoptosis, cytotoxicity, and synergism, coupled with delivery technologies such as nanoparticles, indicating concentrated research attention on overcoming therapeutic resistance through combination strategies and novel drug delivery approaches. The clinical-trial methodology subcluster completes the blue cluster, revealing strong associations between Phase I, Phase II, and Phase III trials, randomized trials, and survival outcomes. This highlights the systematic approach to evaluating the safety and efficacy of drug interactions in oncology, where combination regimens require rigorous clinical validation due to their complexity and the potential for both synergistic therapeutic effects and additive toxicities.

The green cluster reveals a mechanistic research domain organized around three interconnected areas. The cluster is founded on drug metabolism processes, featuring extensive networks that connect cytochrome P450 enzymes, hepatic microsomes, and metabolic pathways, as well as specific enzyme induction and inhibition mechanisms. This emphasizes an understanding of molecular-level interactions. Another major component is transport-protein research, where studies of P-glycoprotein, blood–brain barrier permeability, and drug disposition intersect with natural interaction modulators such as grapefruit juice and St John’s wort. This demonstrates how efflux transporters and herbal products influence drug bioavailability and interaction outcomes. Hepatic safety assessment, which investigates links between hepatotoxicity, liver function monitoring, gene expression profiling and predictive modelling approaches, completes this cluster. This establishes the liver’s central role in evaluating interaction risk and developing computational prediction tools.

The yellow cluster represents HIV therapeutics as a specialized research enclave, where antiretroviral therapy, protease inhibitors, and AIDS-related conditions converge with infected patient populations and specific antiretroviral agents. This clustering pattern underscores the unique pharmacological challenges of HIV treatment, where complex drug interaction profiles between antiretroviral combinations directly influence therapeutic success, viral-resistance patterns, and patient outcomes, requiring dedicated investigation into optimizing multi-drug regimens while navigating the intricate interaction landscape characteristic of HIV pharmacotherapy.

The mechanistic understanding of pharmacokinetic drug–drug interactions evolved substantially throughout our study period. Hirota et al. demonstrated how CYP3A4 inhibitors dramatically increase plasma concentrations of simvastatin, lovastatin, and atorvastatin, while pravastatin and rosuvastatin remain unaffected due to minimal CYP metabolism [51]. This complexity extends to phenoconversion, where drug-induced metabolic changes create mismatches between genetic and observable phenotypes [52]. Clinical implications are evident in the Dutch Pharmacogenetics Working Group guidelines, which document CYP2C19 as poor metabolizers requiring 50% escitalopram dose reductions [53]. Additionally, Tang et al. (2021) showed that fluconazole’s pronounced CYP3A4 inhibition significantly altered fedratinib pharmacokinetics, illustrating the clinical relevance of enzyme-mediated interactions [54].

Our analysis reveals increasing attention to pharmacodynamic interactions affecting therapeutic outcomes through receptor-level mechanisms. Roberti et al. 2021 exemplified this complexity with cenobamate, which, acting through dual mechanisms, inhibited voltage-gated sodium channel persistent currents while modulating GABA(A) receptors at non-benzodiazepine sites [55]. The clinical significance extends to oncology, where Bruin et al. documented exposure–efficacy relationships for talazoparib and exposure–toxicity correlations across poly (ADP-ribose) polymerase inhibitors, predominantly manifesting as hematological adverse events [56]. These findings align with our bibliometric evolution, which showed a progression from documenting isolated receptor interactions to the development of an understanding of integrated pharmacodynamic networks crucial for managing polypharmacy in therapeutic areas with narrow therapeutic windows.

The temporal analysis of citation patterns provides crucial insights into the evolving scientific influence and maturation of drug–drug interaction research across five decades. The Mean Total Citations per Year (MeanTCperYear) metric reveals distinct phases of research impact that closely correspond to, yet uniquely complement, the publication volume trends previously identified. The overall citation trajectory reveals drug–drug interaction research’s transformation from a specialized pharmacological concern to a central pillar of modern pharmaceutical science. The progression from minimal early impact (<1.0) to peak contemporary influence (>3.5) demonstrates the successful integration of DDI considerations into mainstream medical practice, regulatory frameworks, and pharmaceutical development processes. This citation evolution indicates that the field has not only grown in volume but has achieved substantial scientific maturity and practical relevance, with recent research demonstrating an unprecedented influence on clinical practice and pharmaceutical innovation.

The international collaboration patterns in drug–drug interaction research reveal a multipolar research landscape that reflects both traditional pharmaceutical research hierarchies and emerging global partnerships. The United States’ central position, evidenced by its extensive collaboration network and highest TLS, aligns with its historical dominance in pharmaceutical research and regulatory science. However, the emergence of China as a major research contributor, coupled with the formation of distinct regional clusters, suggests a shift toward a more distributed global research architecture. The exceptionally high citation impacts observed in smaller European nations such as Switzerland and Denmark indicate that research quality and international influence are not solely dependent on publication volume but, rather, on specialized expertise and strategic collaborations within established pharmaceutical ecosystems. The four-cluster structure demonstrates how geographic proximity, linguistic similarities, and shared regulatory frameworks continue to shape international research partnerships while also revealing the emergence of transcontinental collaborations that bridge traditional research boundaries. Countries such as Australia and the Netherlands serve as crucial connectors between different regional research communities, facilitating knowledge transfer and methodological standardization across diverse pharmaceutical research traditions. This evolving collaboration landscape reflects the increasingly global nature of drug safety challenges and the recognition that a comprehensive understanding of drug–drug interactions requires diverse perspectives from different healthcare systems, patient populations, and regulatory environments.

The convergence of multiple drug-specific themes (i.e., carbamazepine, fluoxetine) and disease-focused research (i.e., AIDS-related complex) into the methodologically oriented double-blind cluster during 2001–2010 indicates a significant shift toward standardized clinical trial approaches. The remarkable persistence of pharmacokinetics across all three periods, visible as consistent flowing streams in the diagram, underscores its role as the foundational scientific framework of DDI research. Particularly revealing is the transformation of the dominant toxicity cluster into combination therapy research and, subsequently, into in vitro investigations, demonstrating a progression from reactive safety assessments to proactive mechanistic understanding of multi-drug interactions. The emergence of the risk assessment theme in the final period, drawing from multiple previous streams, represents a critical shift toward translational applications, suggesting that the field has evolved from purely mechanistic studies to encompass practical clinical risk-management strategies essential for addressing the complexities of modern polypharmacy.

The temporal evolution of drug–drug interaction research reveals distinct phases characterized by specific therapeutic compounds and a mechanistic understanding. Early research (1990s–early 2000s) concentrated heavily on antiretroviral therapy, with zidovudine and human-immunodeficiency-virus-related interactions dominating the landscape, reflecting the urgent clinical need to understand combination therapy safety during the AIDS epidemic. Concurrently, significant attention was directed toward anticonvulsants such as phenytoin and carbamazepine, known for their potent enzymatic induction properties affecting cytochrome P450 pathways. Cyclosporine, an immunosuppressant with narrow therapeutic windows, and fluoxetine, representing the selective serotonin reuptake inhibitor class, also emerged as critical compounds requiring interaction monitoring. Cancer therapeutics, including cisplatin and 5-fluorouracil, gained prominence in the mid-2000s, highlighting the complexity of managing combination chemotherapy regimens.

The research trajectory demonstrates evolving methodological approaches and expanding clinical considerations based on specific keyword patterns. The coexistence of both “in vivo” (2007, 2012, 2017) and “in vitro” (2009, 2016, 2021) methodologies throughout the timeline, with “in vitro” showing a substantially higher frequency (1237 vs. 245 occurrences), indicates that laboratory-based prediction methods gained prominence alongside clinical studies rather than replacing them. The emphasis on mechanistic understanding is evident through the topics of “human liver-microsomes” (2002, 2009, 2014) and “metabolism” (2005, 2013, 2020) appearing consistently throughout the research period. The emergence of “polypharmacy” (2016, 2020, 2022) as a distinct research focus in recent years directly reflects the growing clinical awareness of multiple-drug-regimen complexities, while “nanoparticles” (2018, 2021, 2023) represents the newest addition to interaction considerations, appearing exclusively in the most recent period. The persistence of “inhibition” (2007, 2016, 2021) across multiple time periods, combined with the consistent presence of “safety” (2012, 2017, 2022) and “risk” (2013, 2019, 2022) in recent years, demonstrates that enzymatic inhibition remains a central mechanistic concern, while risk assessment has become increasingly prioritized in contemporary drug–drug interaction research, as evidenced by the clustering of “management” (2012, 2018, 2022) in the latter portion of the timeline.

DDIs continue to present substantial clinical challenges, necessitating advanced predictive methodologies to improve patient safety and therapeutic outcomes. Recent innovations in computational modeling, such as DDINet, leverage deep sequential-learning architectures combined with attention mechanisms to accurately classify DDIs by their mechanistic pathways, including absorption, metabolism, and excretion processes, thereby potentially reducing the reliance on costly experimental assays [57]. Complementing this, the TSEDDI framework integrates convolutional neural networks with multi-head attention and residual connections to effectively identify toxic side effects arising from drug combinations, underscoring the importance of computational approaches in the early detection of adverse reactions [58].

However, rare, yet severe, DDIs remain difficult to detect due to limited clinical data; addressing this, meta-learning models like RareDDIE utilize biological semantic transferring and dual-granular variational representations to enhance the prediction accuracy in data-scarce scenarios, enabling zero-shot identification and improving drug-synergy evaluations [59]. Furthermore, the timing of drug administration critically influences adverse drug events. The STEM model exemplifies advances in mining real-world datasets to detect DDIs with timing-dependent risks, offering superior false-positive control and increased detection power compared with traditional statistical methods [60].

Beyond conventional pharmacology, investigations into traditional Chinese medicine illustrate the utility of quantitative pharmacology techniques such as the Chou–Talalay method for deciphering complex interactions within multi-compound formulations, providing a framework to optimize efficacy and safety through an understanding of synergistic and antagonistic drug combinations [61]. Future research should focus on integrating these diverse computational and experimental approaches to refine predictive models, expanding databases to include rare DDIs and timing factors, and further exploring substructural drug properties that are linked to toxicity. Such multidisciplinary efforts are vital to advancing personalized medicine and minimizing the clinical risks associated with polypharmacy.

Despite the comprehensive analysis of 19,151 publications spanning five decades, several methodological constraints warrant consideration. The exclusive use of the Web of Science Core Collection, while ensuring data consistency and quality, potentially excludes relevant research published in regional databases or gray literature sources. Additionally, the English language restriction may underrepresent contributions from non-anglophone countries; this could partially explain the lower citation impacts observed for Asian nations compared with Western countries, which may reflect linguistic barriers rather than research quality differences.

The temporal aspects of bibliometric analysis introduce inherent biases, particularly when considering recent publications. Documents from 2020–2025 show artificially low citation rates (0.38–3.89 MeanTCperYear) due to the insufficient time for citation accumulation, potentially underestimating the influence of emerging research areas such as AI or machine learning applications and COVID-19-related drug interactions. Furthermore, although our Python-based thesaurus generator achieved a validation accuracy of 94.3%, automated keyword standardization cannot fully capture the semantic nuances present in the multidisciplinary landscape of DDI research. Although the standardization process is systematic, it may not adequately represent evolving terminology or context-specific meanings in the fields of pharmacology, clinical practice, and regulation.

The period of the COVID-19 pandemic introduces a unique temporal distortion to our dataset. Expedited publication processes and shifted research priorities may affect the thematic distribution and quality assessment of recent publications. This surge may overrepresent certain DDI topics, creating an unsustainable publication trajectory that does not reflect normal research patterns. Additionally, the fractional counting methodology used to calculate country contributions may undervalue nations with extensive international collaborations, which particularly affects smaller countries that predominantly participate in multinational research consortia. While these limitations do not undermine the validity of the study, they do suggest that absolute rankings, the impact of very recent research, and the completeness of global DDI research representation should be interpreted carefully.

Our analysis reveals that the emergence of computational and AI-driven approaches in DDI research offers a chance to democratize access to this important area of study. Traditional DDI research often requires expensive laboratory infrastructure, access to patient populations, and costly clinical trials. However, the recent shift toward computational methods, as evidenced by the emergence of “molecular docking” (2023), “machine learning”, and “in silico” approaches, enables researchers to conduct sophisticated DDI predictions using freely available databases and open-source software. This technological transformation is particularly beneficial for scientists in settings with limited resources, as they can leverage computational tools to conduct meaningful research without the need for extensive physical infrastructure.

Furthermore, the increasing availability of open-access DDI databases (i.e., DrugBank, DDInter), cloud-based computing resources (like Amazon Web Services, Google Cloud Platform, IMB Cloud, and Microsoft Azure), and collaborative platforms reduces traditional barriers to entry. Our collaboration network analysis showing successful partnerships across 100 countries suggests that international collaborations can bridge resource gaps, with computational methods serving as the common platform. This shift toward democratized, computation-based DDI research not only expands the global research community but also ensures that DDI safety profiles are studied across diverse populations and healthcare contexts, ultimately improving pharmaceutical safety worldwide.

## 5. Conclusions

The present large-scale bibliometric and network analysis of 19,151 publications spanning 1975–2025 demonstrates the evolution of DDI research from a narrowly scoped pharmacological and pharmacokinetic inquiry to a fundamental pillar of modern pharmaceutical safety. The field has demonstrated exponential growth, particularly after 2010, with annual publications increasing from 580 to 1194 by 2024, representing a 106% surge. This reflects the integration of DDI considerations into broader discourses on patient safety, personalized medicine, and healthcare system sustainability. The temporal evolution revealed four distinct phases of development, progressing from foundational mechanistic studies to contemporary investigations that incorporate AI, molecular docking, and nanomedicine approaches, as evidenced by the emergence of these terms within the median publication years of 2021–2024.

The thematic evolution analysis revealed a clear transformation in research focus. Initially, research was dispersed and toxicity-focused (1975–2000). Then, there was a methodological consolidation around double-blind trials and pharmacokinetics (2001–2010). Finally, there was a specialization in risk assessment and in vitro studies (2011–2025). The remarkable persistence of pharmacokinetics throughout this period, coupled with its central position in keyword networks, highlights its importance as a scientific foundation. The shift from reactive toxicity assessments to a proactive mechanistic understanding, particularly in oncology, where chemotherapy-related terms form dense clusters, shows how the field is maturing in its approach to complex polypharmacy scenarios.

The present bibliometric analysis provides practical insights for researchers and institutions working in drug interaction science. By mapping the keyword networks of over 100 frequently used terms, we identified important research gaps where basic mechanisms meet clinical practice. The field’s growth is exemplified by leading institutions: the University of Toronto grew from 2 publications in 1979 to 538 by 2025, while the University of Washington reached 390 publications. The recent rise in polypharmacy as a major research topic (456 occurrences; median year 2020), combined with emerging technologies like AI and nanomedicine, highlights the urgent need to address drug safety in aging populations with complex medication regimens.

Several future research directions emerge from the temporal and thematic patterns identified. First, computational and technological approaches show a recent emergence, with “molecular docking” (median year 2023), “nanoparticles” (2021), and “nanomedicine” (2024) representing the newest additions to DDI research terminology. Second, the four-cluster collaboration network, particularly the 43-country Asia–Africa–Middle East cluster demonstrates that research expansion beyond traditional centers in the U.S. and Europe is already underway and could be further strengthened. Third, the thematic evolution from toxicity-focused research (1975–2000) through pharmacokinetics to current themes of risk assessment and in vitro studies (2011–2025) suggests opportunities for better integration between mechanistic and clinical research streams. Fourth, the diverse geographic participation revealed in our analysis from established leaders to emerging contributors—like China’s significant increase since 2015—indicates the value of investigating regional differences in DDI patterns. Finally, the keyword evolution showing “polypharmacy” (2020), “management” (2018), and “risk” (2019) as recent high-frequency terms points to an increasing focus on practical clinical applications and real-world medication safety.

This analysis offers strategic insights that extend beyond bibliometric evaluation, highlighting opportunities for international collaboration, informed research prioritization, and translational impact across clinical domains. As polypharmacy becomes a defining feature of contemporary medicine, these findings underscore the need for integrative, forward-looking approaches capable of supporting safer and more effective therapeutic practices worldwide.

## Figures and Tables

**Table 1:** Global contributions to drug–drug interaction research (1975–2025).

Country	Documents	Citations	Average Citations/Document	TLS
USA	7001	287,816	41.11	2610
China	1723	39,372	22.85	690
United Kingdom	1404	56,468	40.22	1596
Italy	1124	37,660	33.51	975
Germany	1045	39,643	37.94	1054
Canada	846	39,188	46.32	678
France	819	28,757	35.11	828
India	818	14,501	17.73	401
Australia	682	26,241	38.48	592
Japan	664	16,811	25.32	356

TLS, total link strength.

**Table 2:** Leading publication sources in drug–drug interaction research (1975–2025).

Source	h-Index	g-Index	m-Index	Total Citations	Publications	Publication Start
*Clinical Pharmacokinetics*	71	136	1.449	18,829	158	1977
*Journal of Clinical Oncology*	58	92	1.381	12,632	92	1984
*Drug Safety*	51	83	1.457	7610	131	1991
*Clinical Cancer Research*	49	79	1.633	7020	123	1996
*Annals of Pharmacotherapy*	47	74	1.382	8855	274	1992
*Drugs*	46	89	1.314	8092	89	1991
*Epilepsia*	45	79	0.957	6710	106	1979
*American Journal of Health-System Pharmacy*	44	71	1.419	6660	190	1995
*Clinical Pharmacology &Therapeutics*	44	78	0.898	6312	100	1977
*British Journal of Clinical Pharmacology*	42	72	0.977	5949	145	1983

h-index, Hirsch index; g-index, Egghe’s g-index; m-index, m-quotient.

**Table 3:** Top-cited publications in drug–drug interaction research (1975–2025).

Paper/Source	Title	TC	TC/Year	Normalized TC	DOI
Gaede P, 2008, *N Engl J Med*	Effect of a multifactorial intervention on mortality in Type 2 diabetes	2534	140.78	47.03	10.1056/NEJMoa0706245
Goldberg RM, 2004, *J Clin Oncol*	A randomized controlled trial of fluorouracil plus leucovorin, irinotecan, and oxaliplatin combinations in patients with previously untreated metastatic colorectal cancer	1876	85.27	31.41	10.1200/JCO.2004.09.046
Carneiro BA, 2020, *Nat Rev Clin Oncol*	Targeting apoptosis in cancer therapy	1738	289.67	74.54	10.1038/s41571-020-0341-y
Law V, 2014, *Nucleic Acids Res*	DrugBank 4.0: shedding new light on drug metabolism	1611	134.25	42.59	10.1093/nar/gkt1068
Einhorn LH, 1977, *Ann Intern Med*	Cis-diamminedichloroplatinum, vinblastine, and bleomycin combination chemotherapy in disseminated testicular cancer	1486	30.33	11.34	10.7326/0003-4819-87-3-293
Scott IA, 2015, *JAMA Intern Med*	Reducing inappropriate polypharmacy: the process of deprescribing	1021	92.82	28.65	10.1001/jamainternmed.2015.0324
Ashley CE, 2011, *Nat Mater*	The targeted delivery of multicomponent cargos to cancer cells by nanoporous particle-supported lipid bilayers	887	59.13	21.34	10.1038/NMAT2992
Gnjidic D, 2012, *J Clin Epidemiol*	Polypharmacy cutoff and outcomes: five or more medicines were used to identify community-dwelling older men at risk of different adverse outcomes	847	60.50	21.78	10.1016/j.jclinepi.2012.02.018
Galati G, 2004, *Free Radic Biol Med*	Potential toxicity of flavonoids and other dietary phenolics: significance for their chemopreventive and anticancer properties	847	38.50	14.18	10.1016/j.freeradbiomed.2004.04.034
Lynch T, 2007, *Am Fam Physician*	The effect of cytochrome P450 metabolism on drug response, interactions, and adverse effects	792	41.68	15.68	-

TC, total citations; DOI, digital object identifier.

## Data Availability

The raw data supporting the conclusions of this article will be made available by the authors on request.

## Abbreviations

The following abbreviations are used in this manuscript:

ADR	Adverse drug reactions
AI	Artificial intelligence
COVID-19	Coronavirus disease 2019
DDI	Drug–drug interaction
pDDI	Potential drug–drug interaction
TLS	Total link strength
